# Ketamine-Induced Syndrome of Inappropriate Antidiuretic Hormone Secretion and Hyponatremia

**DOI:** 10.7759/cureus.25931

**Published:** 2022-06-14

**Authors:** Kelash Kumar, FNU Poonam, Teesha Rani, FNU Prinka, Cece E Ibeson, Ifeanyi Nwosu, Vijay Shetty, Anthony N Kalloo

**Affiliations:** 1 Department of Internal Medicine, Maimonides Medical Center, Brooklyn, USA; 2 Department of Internal Medicine, Wyckoff Heights Medical Center, Brooklyn, USA; 3 Department of Medicine, Ziauddin University, Karachi, PAK; 4 Department of Internal Medicine, Maimonides Medical Center, New York City, USA

**Keywords:** ketamine-associated cystitis, drug addiction, syndrome of inappropriate antidiuretic hormone secretion, ketamine, hyponatremia

## Abstract

Ketamine is a dissociative anesthetic commonly used for the induction and maintenance of anesthesia and has a well-known role in analgesia. However, it also has the potential for addiction, which can lead to neurological, psychological, systemic, and biochemical consequences. In this case report, we are highlighting a rare case of a young Asian female with Ketamine addiction who presented with urinary complaints. The patient was found to have hyponatremia and laboratory tests were consistent with a syndrome of inappropriate antidiuretic hormone (SIADH) release in the absence of other causes.

## Introduction

Ketamine is a noncompetitive antagonist of the N-methyl-d-aspartate (NMDA) receptor [1]. It is distinct from other general anesthetics and drugs used for sedation and analgesia as it causes a trance-like cataleptic state characterized by profound unconsciousness, amnesia, deep analgesia with retention of ocular, protective airway reflexes, and cardiopulmonary stability [2]. Ketamine is also famous as a dissociative anesthetic. Ketamine is available in liquid and powder form and is being abused by many individuals [3]. Because of its reinforcing out-of-body experiences and rewarding properties, Ketamine has become a recreational drug, particularly in the context of raves, and its recreational use has grown progressively worldwide in the past few decades [4].

## Case presentation

A 31-year-old Asian female with a past medical history of Ketamine abuse and hemorrhagic cystitis presented with progressive difficulty in urination for one week. It was associated with painful urination, increased frequency, and lower abdominal pain, which gets worse at the time of urination. The patient denied fever, chills, nausea, vomiting, blood, pus or foul smell in urine, headache, dizziness, or lethargy. The patient is well known to our facility and has been seen multiple times for a similar problem. In her last admission, she was diagnosed to have Ketamine induced hemorrhagic cystitis. Of note, the patient has been addicted to Ketamine for a long period. The patient denied using other medications and recreational substances.

Vitals: heart rate 115/min, blood pressure 115/91 mmHg, respiratory rate 18/min temperature 98.8F. Physical exam was notable for a thin lean female, lying on the bed in no acute distress, mild supra-pubic tenderness noted. No crackles on lung auscultation, abdominal distention, costovertebral angle tenderness, or pedal edema were noted.

Pertinent laboratory studies (Table 1) showed white blood cell (WBC) count 11.8 k/µL, serum sodium 121 mmol/L, serum potassium 4.7 mmol/L, blood urea nitrogen (BUN) 22.22 mg/dL, creatinine 1.0 mg/dL, serum osmolality 270 mosmol/kg, thyroid-stimulating hormone (TSH) 2.16 mciu/L, random cortisol 25 µg/dL, urine sodium 76 mmol/L, urine chloride 59 mmol/L and urine osmolality 425 mosm/kg. Urinalysis was consistent with urinary tract infection WBC > 50/hpf, high leukocyte esterase, and moderate abundant bacteria in the presence of symptoms. However, the red blood cell count was 2-5 cells/hpf.

**Table 1:** Laboratory results of the patient

Test	Result	Reference range
White Blood Cell (WBC) Count	11.8 k/µL	4.8-10.8 k/µL
Serum Sodium	121 mmol/L	135-145 mmol/L
Serum Potassium	4.7 mmol/L	3.4-4.8 mmol/L
Blood urea nitrogen (BUN)	22.22 mg/dL	7-21 mg/dL
Creatinine	1.0 mg/dL	0.3-1.1 mg/dL
Serum Osmolality	270 mosmol/kg	275-295 mosmol/kg
Thyroid stimulating hormone (TSH)	2.16 mciu/L	0.39-4.08 mciu/L
Random Cortisol	25 µg/dL	6.7-27.6 µg/dL
Urine Sodium	76 mmol/L	20-40 mmol/L
Urine Chloride	59 mmol/L	20-40 mmol/L
Urine Osmolality	425 mosm/kg	500-800 mosm/kg

The patient was started on Ceftriaxone, Pyridium (Phenazopyridine) for symptomatic relief of urinary symptoms and fluid restriction <1 L/day. The patient’s presenting symptoms resolved, and serum sodium level improved to 124 mEq/L and 128 mEq/L in a couple of days. Nothing grew in the urine culture. The patient was discharged on Pyridium as needed, water restriction less than 1 L/day, advised to quit Ketamine use and outpatient follow up with primary care provider for continuity of care.

## Discussion

Ketamine is a dissociative anesthetic, which means that it distorts the perception of sight and sound while producing a feeling of detachment from one's self and surroundings [3]. There are several street names for Ketamine such as Special K, Green K, Super K, Super acid, Jet, and Cat Valium [3]. It was originally synthesized at Parke-Davis Company in 1962 by Alvin Stevens and was first used in humans by Corssen and Domino in 1965 [3]. Ketamine rapidly crosses the blood-brain barrier and is primarily thought to act as a noncompetitive inhibitor of the N-methyl-d-aspartate (NMDA) receptor [5]. It produces a trance-like cataleptic state characterized by profound analgesia and amnesia, with retention of ocular reflexes, protective airway reflexes, spontaneous respirations, and cardiopulmonary stability, hence also preferred in emergency and acute care settings [2]. The above properties make it distinct from other general anesthetics and drugs used for sedation and analgesia. It can be effective in managing non-responsive pain and complex regional pain syndrome (CRPS) [6].

Ketamine can be administered by intravenous, intramuscular, snorting, and smoking routes. The predominant route of ketamine administration for recreational use is intranasal and rarely intravenously [4]. Addiction is defined as being a primary chronic disease of the brain reward, motivation, and memory in this complex circuitry [3]. Reports of out-of-body experiences have been consistently associated with recreational use and abuse of Ketamine, which has grown steadily worldwide in the past few decades [4].

Reported adverse effects of Ketamine include tachycardia, hypertension, anxiety, dysphoria, nightmares, hallucinations, disorientation, insomnia, euphoria agitation, blurred vision, sedation, and elevation of liver enzymes [4-6].

Ketamine is mainly excreted via urine 90% in the form of metabolites with an elimination half-life of about 2.5 hours. Ketamine is expected to be completely cleared from adult circulation within 12.5-17.5 hours. The older age, low body mass, and impaired renal function of this patient may prolong the elimination of Ketamine [6]. Limited literature is available that describes the rare and unusual association between Ketamine and syndrome of inappropriate antidiuretic hormone (SIADH) [6].

The release of antidiuretic hormone is complex a process involving serum sodium, serum osmolality, and the interaction of afferent inputs with local and intrinsic mechanisms in the regulation of magnocellular neurosecretory responses to physiological challenges [4]. However, antidiuretic hormone release can also be related to pharmacological and non-pharmacological factors (Table 2) that can centrally increase the release of ADH like antidepressants (tricyclics, selective serotonin reuptake inhibitors, and monoamine oxidase inhibitors) and antipsychotic drugs (phenothiazines and butyrophenones) [4].

**Table 2:** List of pharmacological and non-pharmacological factors which stimulates antidiuretic hormone secretion [4]. MAO = monoamine oxidase; MDMA = 3,4-methylenedioxymethamphetamine; NSAIDs = nonsteroidal antiinflammatory drugs; SIADH = syndrome of inappropriate antidiuretic hormone secretion

Category	Pharmacologic and nonpharmacologic risk factors
Antidepressants	Selective serotonin reuptake inhibitor, Tricyclic antidepressants, MAO inhibitors, Venlafaxine
Anticonvulsants	Carbamazepine, Sodium valproate, Lamotrigine
Antipsychotics	Phenothiazines, Butyrophenones
Anticancer drugs	Vinca alkaloids, Platinum compounds, Ifosfamide, Melphalan, Cyclophosphamide, Methotrexate, Pentostatin
Antidiabetic drugs	Chlorpropamide, Tolbutamide
Vasopressin analogues	Desmopressin, Oxytocin, Terlipressin, Vasopressin
Miscellaneous drugs	Opioids, MDMA, Levamisole, Interferon, NSAIDs, Clofibrate, Nicotine, Amiodarone, Proton pump inhibitors, Monoclonal antibodie, Linezolid, Diuretics
Patient factors	Age over 65 years, Acute pain, Pneumonia, Malignancies, Surgery, Hypothyroidism, Hereditary

The antidiuretic hormone acts at the renal collecting tubules, where it inhibits renal excretion and promotes the reuptake of water into the vascular system. This increases total body water (TBW) and blood volume, diluting serum sodium concentrations. As a physiologic reflex, the increase in TBW transiently promotes urinary sodium excretion in an attempt to normalize the extracellular volume and equilibrate sodium concentration gradients. This reflexive process further reduces plasma sodium concentration and causes hyponatremia [6].

The patient described in our case report is a young Asian female with a history of Ketamine addiction, who was hyponatremia (defined as a serum sodium level less than 135 mmol/L) on admission. No evidence of dehydration or volume overload was found during clinical evaluation. Hyponatremia improved on avoidance of Ketamine and water restriction. On our assessment, the results satisfied the 1967 Bartter and Schwartz diagnostic criteria for SIADH, namely evidence of hyponatremia with corresponding hypo-osmolarity, continued renal excretion of sodium, and no evidence of volume depletion, and subsequent correction of hyponatremia by fluid restriction. Alternate causes of hyponatremia including hepatic disease, cardiac failure, adrenal, and hypothyroidism were absent [6]. Naranjo score of 9 was calculated, suggesting that the correlation between ketamine and hyponatremia was definitive [6]. Hence, a diagnosis of ketamine-induced SIADH was made. It is proposed that Ketamine also centrally stimulates the release of antidiuretic hormone from the hypothalamus [6]. We believe this pathophysiologic mechanism was responsible for precipitating SIADH in this case.

## Conclusions

Our case is of clinical significance for providers who use Ketamine for sedation and pain management and also for hospitalists and internists who encounter patients with Ketamine addiction or abuse potential. Ketamine use could result in SIADH and possible synergistic effects when different drugs that potentiate SIADH are combined. By being aware of this effect, providers can develop a better appreciation and awareness, and they should monitor, detect, and manage as appropriate.
